# Mebendazole inhibits PELI3-mediated ubiquitination of TRADD in non-small cell lung cancer cells

**DOI:** 10.3389/fonc.2025.1728170

**Published:** 2026-01-09

**Authors:** Fannian Li, Xiaoning Li, Haitao Li, Shuai Li, Yanchao Liu, Xianhua Bai, Tianjie Qi, Xiumin Zhao, Yuzheng He

**Affiliations:** 1 Department of Thoracic Surgery, the First Affiliated Hospital of Xing Tai Medical College, XingTai, Hebei, China; 2 Department of Thoracic Surgery, Hebei General Hospital, Shijiazhuang, Hebei, China; 3 The First Department of Pulmonary and Critical Care Medicine, the Second Hospital of Hebei Medical University, Hebei Key Laboratory of Respiratory Critical Care Medicine, Hebei Institute of Respiratory Diseases, Shijiazhuang, Hebei, China; 4 The Second Department of Pulmonary and Critical Care Medicine, the Second Hospital of Hebei Medical University, Shijiazhuang, Hebei, China; 5 Department of Medical Imaging, the Second Hospital of Hebei Medical University, Shijiazhuang, Hebei, China; 6 Department of the Integrated Treatment of Traditional Chinese and Western Medicine, the Second Hospital of Hebei Medical University, Shijiazhuang, Hebei, China; 7 Department of Thoracic Surgery, the Second Hospital of Hebei Medical University, Shijiazhuang, Hebei, China

**Keywords:** mebendazole, PELI3, TRADD, E3 ubiquitin ligase, non-small cell lung cancer

## Abstract

**Introduction:**

Tumor necrosis factor receptor 1-associated death domain protein (TRADD) can trigger proapoptotic autophagy in non-small cell lung cancer (NSCLC). While the potential ubiquitin-protein ligase (E3) against TRADD is not well deciphered.

**Methods:**

UbiBrowser was used to predict the potential E3 ubiquitin ligase to bind with TRADD. Co-immunoprecipitation was performed in HEK293T cells co-transfected with Myc-PELI3, Flag-TRADD, or HA-Ub plasmids. Increasing doses of Myc-PELI3 were transfected into HCC827 and A549 cells, and the relative expression of TRADD was detected. Cycloheximide chase assay was performed in A549 cells transfected with Myc-PELI3 plasmids, and the stability of TRADD was revealed. CCK-8 assay was performed in A549 and HCC827 cells incubated with increasing doses of Mebendazole. The expression of TRADD and PELI3 after Mebendazole incubation was assayed with Western Blot and RT-PCR. The potential E3 ubiquitin ligase of PELI3 was predicted by the UbiBrowser platform, and the binding of PELI3 with TRADD was testified in HEK293T cells co-transfected with Myc-PELI3 and Flag-TRADD plasmids.

**Results:**

PELI3 overexpression diminished the relative protein expression of TRADD, while not affecting the relative mRNA expression in both A549 and HCC827 cells. Cycloheximide assay and following HA-Ub detection demonstrated that PELI3 decreased the protein stability of TRADD by inducing polyubiquitination. Mebendazole inhibited the viability of HCC827 and A549 cells with diminished expression of PELI3 and increased expression of TRADD.

**Conclusions:**

PELI3 can function as an E3 ubiquitin ligase to ubiquitinate TRADD, and Mebendazole might be a promising drug to affect PELI3 expression in NSCLC.

## Introduction

As the primary cause of cancer-related deaths, lung cancer demonstrates the highest mortality rates in both women and men (1, 2). Non-small-cell lung cancer (NSCLC) is often diagnosed at advanced stages and accounts for over 80% of lung cancer. The increasing incidence and mortality of NSCLC pose an immense burden to public health challenges (3). NSCLC is primarily resected with curative intent and is not sensitive to adjuvant chemotherapy when compared with small-cell lung cancer (4).

More than 60% of NSCLC patients possess epidermal growth factor receptor (EGFR), which can be targeted with EGFR tyrosine kinase inhibitors. Although EGFR-targeted therapy is the preferential option for advanced NSCLC patients, molecular targets display compromised effects due to significant drug resistance (5, 6). Mechanically, EGFR treatment could rapidly upregulate tumor necrosis factor (TNF) expression and the assembly of antiapoptotic, nuclear factor kappa B (NF-κB)-inducing (TNF-R1–TRADD–RIP/TRAF2) receptor complexes, which contributes to primary and secondary resistance to EGFR inhibition (7, 8). It is worth noting that, the TNF-R1-associated death domain protein (TRADD) is also involved in the pro-apoptotic receptor complexes (TNF-R1–TRADD–FADD) to mediate apoptosis (9).

Although the disassembly and regulation mechanisms of TNF-R1 complexes are still unclear, the mechanism that can transmit the antiapoptotic effect to the apoptotic effect will be an interesting field in NSCLC research. Previous research demonstrates that antifungal Sertaconazole can stabilize TRADD from ubiquitination-mediated degradation, which further triggers TRADD-dependent proapoptotic autophagy in NSCLC cells (10). All of these indicate that TRADD ubiquitination research might be a novel therapeutic option for NSCLC patients. Mebendazole was selected because, like Sertaconazole, it has demonstrated antitumor activity and the ability to influence apoptosis-related pathways (11). As a repurposed anthelmintic with a strong safety profile, Mebendazole has shown potent efficacy across multiple solid tumors, including NSCLC (12). Its reported capacity to disrupt ubiquitin-dependent processes makes it a suitable candidate for exploring whether TRADD stabilization can be enhanced by targeting its upstream regulators. These features support our rationale for investigating whether Mebendazole modulates the PELI3/TRADD axis.

Ubiquitination is a three-step process involving three enzymes, such as ubiquitin-activating enzyme (E1), ubiquitin-conjugating enzyme (E2), and ubiquitin-protein ligase (E3) (13). E3 ubiquitin ligase recruits substrates, promotes ubiquitin transfer onto targets, and induces non-degradative signaling or proteasomal degradation signaling (14). Pellino E3 ubiquitin protein ligase family member 3 (PELI3) is initially found to interact with interleukin-1 receptor-associated kinase (IRAK) with E3 ubiquitin ligase activity (15). In this investigation, based on the UbiBrowser platform, the binding of PELI3 with TRADD is identified, which is further testified with co-immunoprecipitation, cycloheximide chase assay, and polyubiquitination assay. Mebendazole is testified to inhibit the viability of NSCLC and down-regulate the relative expression of PELI3, with the up-regulation of TRADD.

## Materials and methods

### Ubiquitin ligases prediction

To screen the potential E3 ubiquitin ligases of TRADD, the online platform UbiBrowser 2.0 (http://ubibrowser.bio-it.cn/ubibrowser_v3/) was utilized to predict the ubiquitin ligase-substrate interactions (16). All parameters and options were in their default form.

### Plasmid and siRNA transfection

The complete human TRADD or PELI3 coding region was amplified by PCR, and ligated into pcDNA3.1-Flag vector or pcDNA3.1+/C-Myc vector (NovoPro), which was further transfected into HEK293T, A549, and HCC827 cells (National Biomedical Experimental Cell Resource Bank of China) with Lipofectamine 2000 (Thermo Fisher Scientific). The PCR primers utilized were listed: TRADD, forward primer, 5’-CGGGATCCATGGCAGCTGGGCAAAAT-3’, reverse primer, 5’- CCCTCGAGCTAGGCCAGGCCGCCATT-3’; PELI3, forward primer, 5’- CTACTCGAGGCTCCCTGGGGCCCCC-3’, reverse primer, 5’- CTAGCGGCCGCTTCTGGAGAGTGCTCAATGGA‐3’. SiRNA targeting PELI3 was synthesized by GenePharm, Suzhou, China (target sequence, 5’-GAGGACAGACTGTTA ACAAAT-3’), which was further transfected into A549 cells with Lipofectamine 2000.

### CCK-8 assay

Cell viability was assayed with a Cell-Counting Kit-8 (CCK-8, Beyotime). A549 and HCC827 cells were incubated with increasing concentrations of Mebendazole (0, 2, 4, 8 μM) in 96-well for 24 h, and 10 μl CCK-8 solution was added and incubated for 2 h at 37°C. The absorbance was assayed with Molecular Devices SpectraMax Plus 384 Microplate Reader.

### Quantitative RT‐PCR analysis

TRIzol was utilized to extract total RNA from Myc-PELI3 plasmids transfected A549 and HCC827 cells or Mebendazole-incubated A549 and HCC827 cells, which was further reverse transcribed into cDNA with the PrimeScript RT reagent Kit (Takara). SYBR Green Supermix (Bio‐Rad) was used to reveal the amplification of interest genes in a CFX96 Real-Time System (Bio‐Rad). The reaction procedures were as follows: 95 °C, 8 min; 95 °C, 15 s, 35 cycles; 60 °C, 45 sec. Expression data was normalized to GAPDH mRNA expression. The primer pairs: TRADD, forward 5’-TTCTGCGGCTATTGCTGA-3’, reverse 5’-TGAAACTGTAAGGGCTGG-3’; PELI3, forward 5’-CTGGAAGGAAACCCTGAAGT-3’, reverse 5’-AGCGGCGTGGAGATGTG-3’; GAPDH, forward 5’-ACAACTTTGGTATCGTGGAAGG-3’, reverse 5’-GCCATCACGCCACAGTTTC-3’.

### Co-immunoprecipitation

To perform a Co-immunoprecipitation (Co-IP) assay, Pierce Classic IP Kit (Thermo Scientific) was utilized as indicated by the manufacturer. Myc-PELI3 or Flag-TRADD plasmids transfected HEK293T cells were lyzed in IP Lysis buffer (Thermo Scientific) with protease inhibitor cocktail (Roche) and centrifuged (13,000 g, 10 min) to get the supernatant, which was incubated with designated antibody (2  μg, overnight, 4 °C). The supernatant was further incubated with Protein A/G Plus Agarose for 1 h and detected with immunoblotting analysis.

Then, protein lysates were separated by SDS/PAGE and transferred onto polyvinylidene difluoride membranes. The membranes were incubated overnight with primary antibodies against TRADD, PELI3, Myc, and GAPDH, and a chemiluminescence assay was performed with the GE Healthcare ECL system.

### Cycloheximide chase assay

Cycloheximide chase assay was utilized to detect kinetical protein degradation with immunoblotting analysis. Myc-PELI3 plasmids transfected A549 cells were incubated with 100 μg/ml Cycloheximide for 0, 4, 8, and 16 h. Immunoblotting analysis against TRADD and GAPDH was utilized to reveal TRADD degradation kinetics.

### Ubiquitination assay

Myc-PELI3, HA-Ub-K48, or Flag-TRADD plasmids were transfected into HEK293T cells for 48 h before the collection of cell lysates. The supernatant was co-immunoprecipitated with anti-Flag antibody and protein G-agarose, which was then incubated in an SDS-loading buffer (95 °C, 5 min) and subjected to mouse anti-HA (BioLegend) antibody.

### Statistical analysis

Differences between two groups were analyzed using the unpaired Student’s t-test. Comparisons among multiple groups were performed using one-way or two-way ANOVA. The significance level was set at a *p-value* < 0.05. All statistical analyses were performed using GraphPad Prism.

## Results

### PELI3 can bind with TRADD

**Figure 1:**
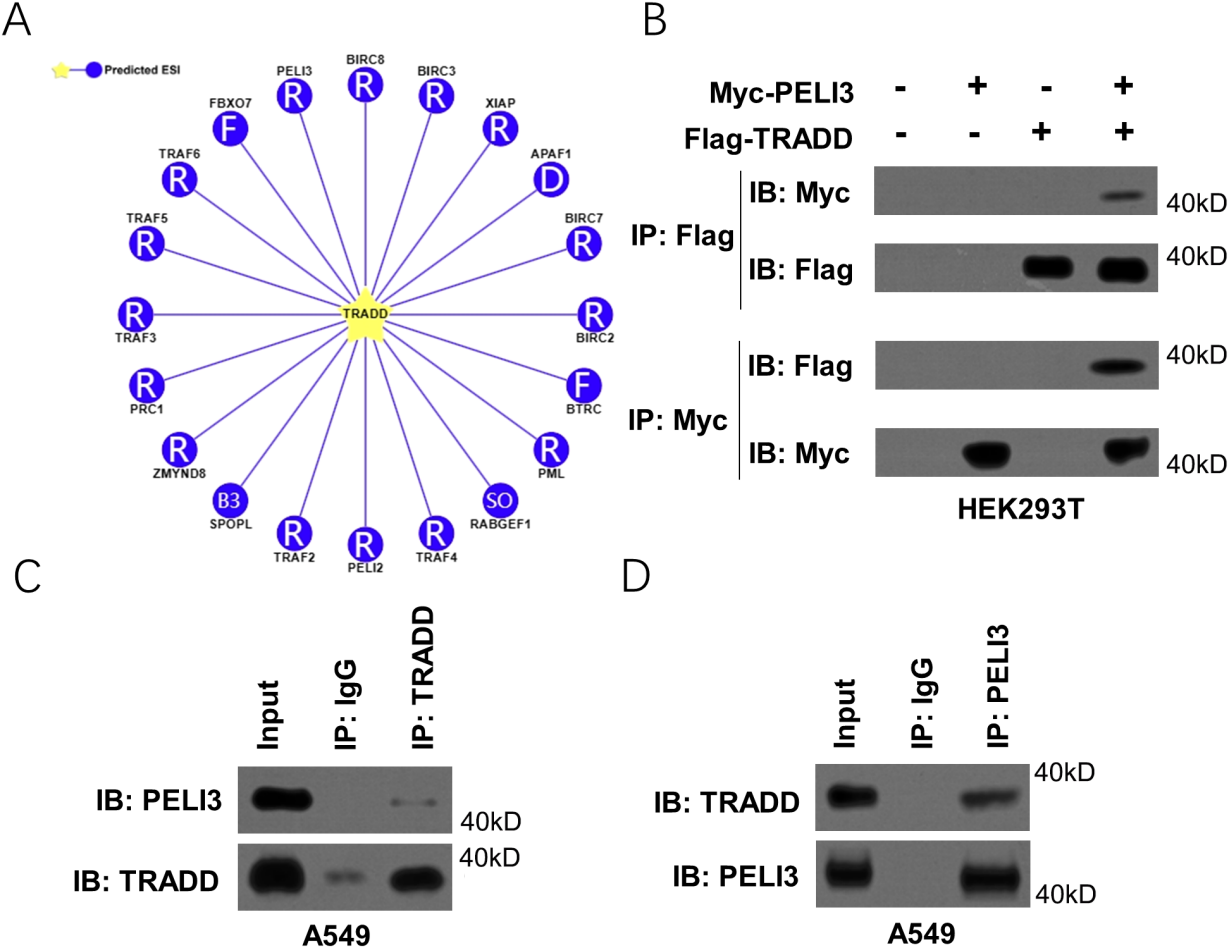
PELI3 binds to TRADD in NSCLC cells. **(A)** E3 ligases of TRADD were predicted by UbiBrowser2.0 online (http://ubibrowser.bio-it.cn/ubibrowser_v3/). **(B)** Myc-PELI3 or Flag-TRADD plasmids were transfected into HEK293T cells for 48 hours, followed by co-immunoprecipitation (Co-IP) with an anti-Myc or anti-Flag antibody. For the Co-IP assays, each IP group was performed using an equal amount of the specified tag antibody (anti-Myc or anti-Flag) for precipitation. **(C, D)** A549 cells were lysed for co-immunoprecipitation with an anti-TRADD or anti-PELI3 antibody.

To screen the potential E3 ubiquitin ligases to ubiquitinate TRADD, UbiBrowser, an online platform to predict ubiquitin ligase-substrate interactions, was utilized. As shown in **Figure 1A**, 20 potential E3 ubiquitin ligases of TRADD were predicted. Through further literature review and co-immunoprecipitation assay in HEK293T cells co-transfected with Myc-PELI3 and Flag-TRADD plasmids, we found that only PELI3 strongly bound to TRADD (**Figure 1B**). Moreover, co-immunoprecipitation assay in A549 cells also confirmed the binding of PELI3 with TRADD with an anti-TRADD or anti-PELI3 antibody (**Figures 1C, D**).

### PELI3 inhibits the protein expression of TRADD in NSCLC cells

**Figure 2:**
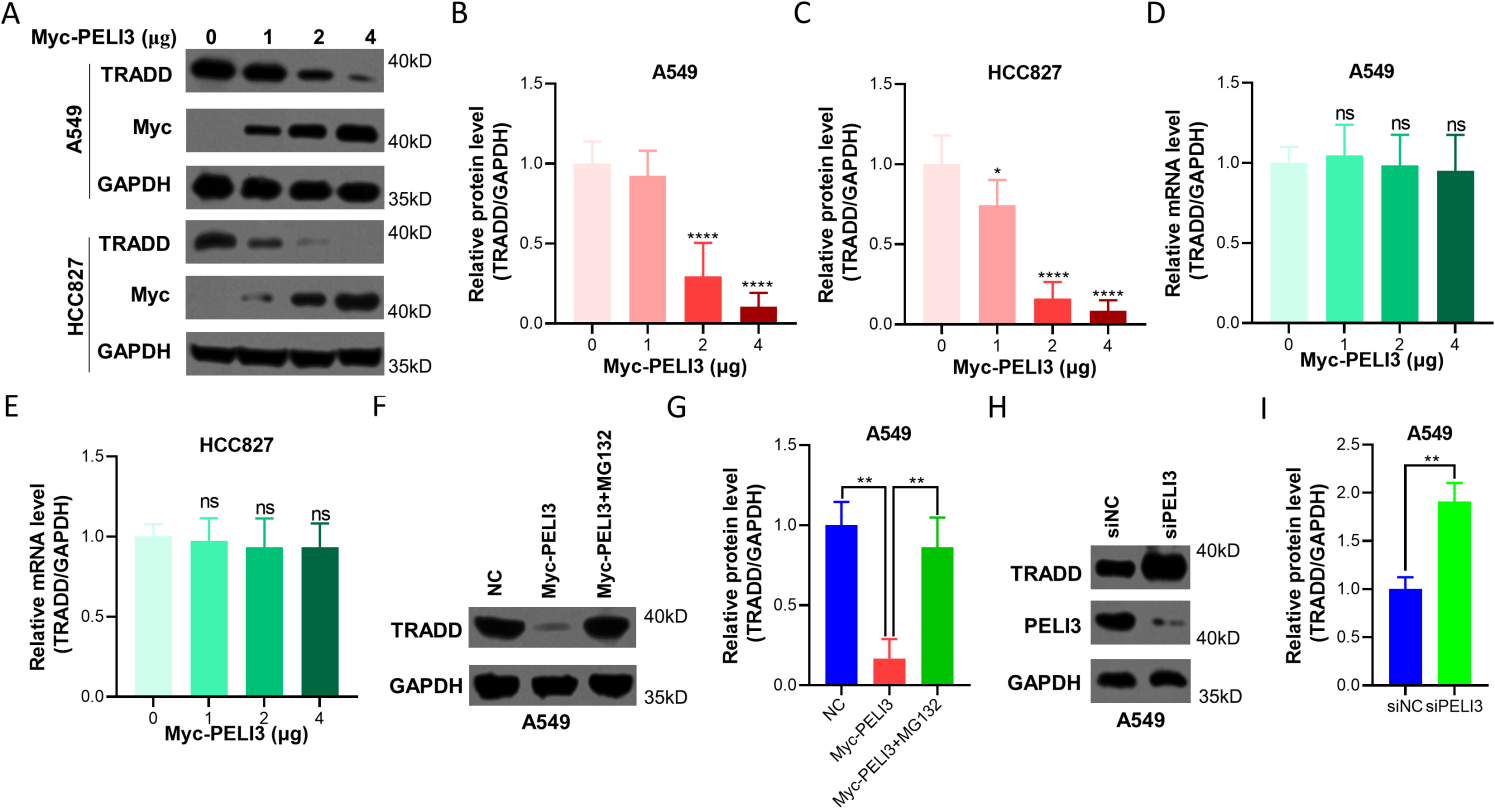
Overexpression of PELI3 inhibits the protein expression of TRADD in NSCLC cells. **(A)** Increasing doses of Myc-PELI3 plasmids were transfected into A549 and HCC827 cells for 72 hours, followed by immunoblotting analysis against TRADD, Myc, and GAPDH. For all transfection experiments, the total amount of DNA was kept constant by supplementing with empty vector plasmids. The ‘0 μg’ Myc-PELI3 condition represented transfection with 4 μg of empty vector. Optical density analysis of TRADD from Figure A in A549 **(B)** and HCC827 cells **(C)**. Above cells were also prepared for RT-qPCR analysis to detect the relative mRNA levels of TRADD in A549 **(D)** and HCC827 cells **(E)**. GAPDH was used as an internal control. A549 cells transfected with empty vector or Myc-PELI3 plasmids were incubated with 5 μM MG132for 24 hours, followed by immunoblotting analysis **(F)**, and optical density of TRADD was also measured **(G)**. A549 cells were transfected with indicated siRNAsfor 72 hours, followed by immunoblotting analysis **(H)**, and optical density of TRADD was also measured **(I)**. ^*^p < 0.05; **p < 0.01; ^****^p < 0.0001; ns means not significant.

To decipher the potential role of PELI3 on TRADD, Myc-PELI3 plasmids were transfected into A549 and HCC827 cells to construct overexpression models (**Figure 2A**), Decreased TRADD protein expression was observed in PELI3 over-expressed A549 (**Figure 2B**) and HCC827 cells (**Figure 2C**) in a dose-dependent manner. On the other hand, over-expressed PELI3 did not affect the relative mRNA expression of TRADD in A549 (**Figure 2D**) and HCC827 cells (**Figure 2E**). Moreover, the proteasome inhibitor MG132 could significantly rescue PELI3-driven downregulation of TRADD in NSCLC cells (**Figures 2F, G**). In contrast, knockdown of PELI3 significantly increased TRADD in NSCLC cells (**Figures 2H, I**).

### PELI3 decreases the protein stability of TRADD by inducing its polyubiquitination

**Figure 3:**
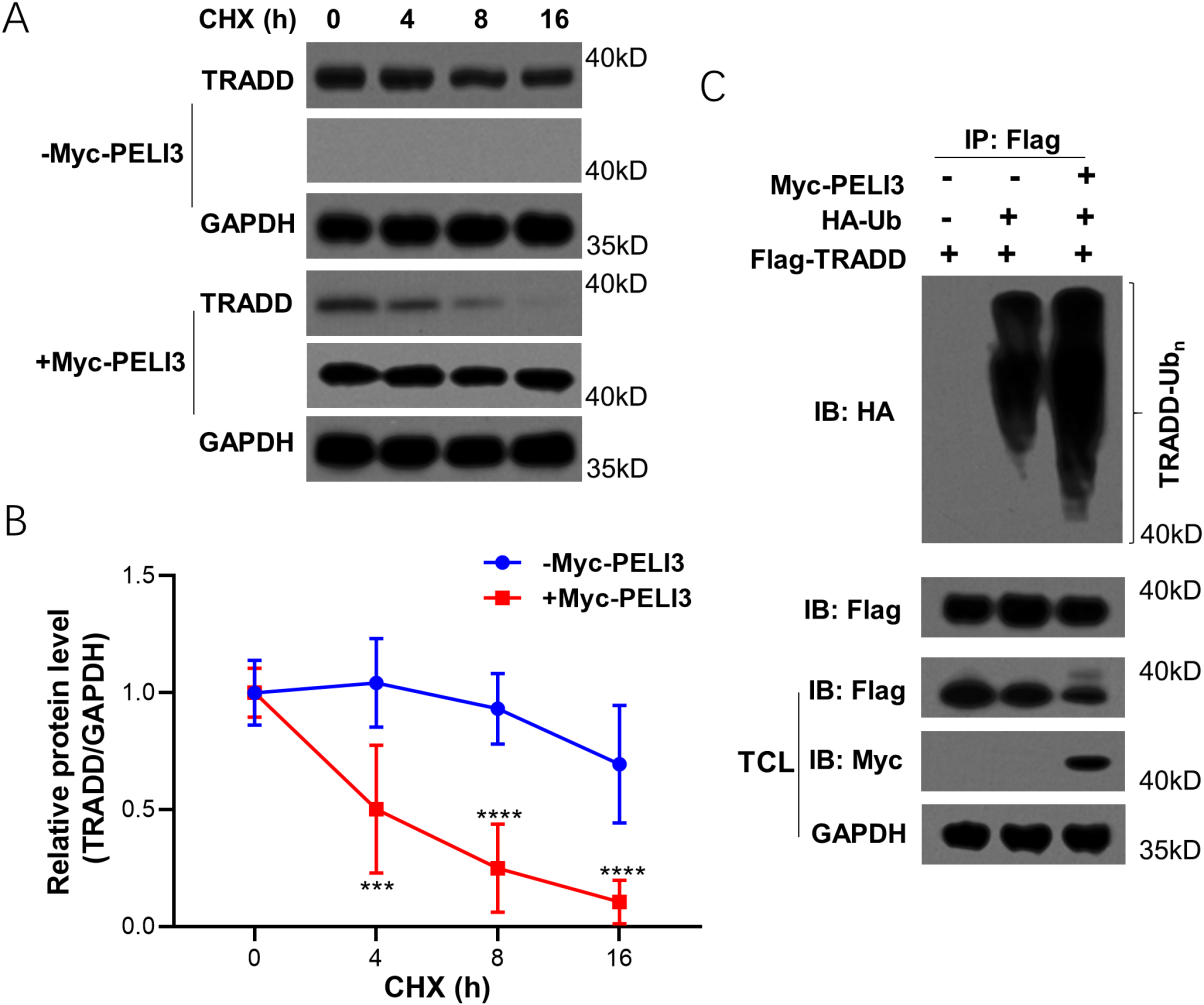
Overexpression of PELI3 decreases the protein stability of TRADD by inducing its polyubiquitination. Empty vector or Myc-PELI3 plasmids were transfected into A549 overnight, incubated with 100 μg/ml Cycloheximide (CHX) for indicated times, and followed by immunoblotting analysis against TRADD, Myc tag and GAPDH **(A)**. Optical density analysis was conducted **(B)**. “-Myc-PELI3” refers to cells transfected with an empty vector. **(C)** Myc-PELI3, HA-Ub-K48, or Flag-TRADD plasmids were transfected into HEK293T cells for 48 hours. Cells were lysed for co-immunoprecipitation with an anti-Flag antibody. ^***^p < 0.001; ^****^p < 0.0001.

Cycloheximide chase assay was performed in PELI3 over-expressed A549 (**Figure 3A**), and PELI3 overexpression could significantly shorten the protein half-life of TRADD (**Figure 3B**). Ubiquitination assay also demonstrated that PELI3 can significantly promote the polyubiquitination of TRADD (**Figure 3C**).

### Mebendazole inhibits PELI3 expression in NSCLC cells

**Figure 4:**
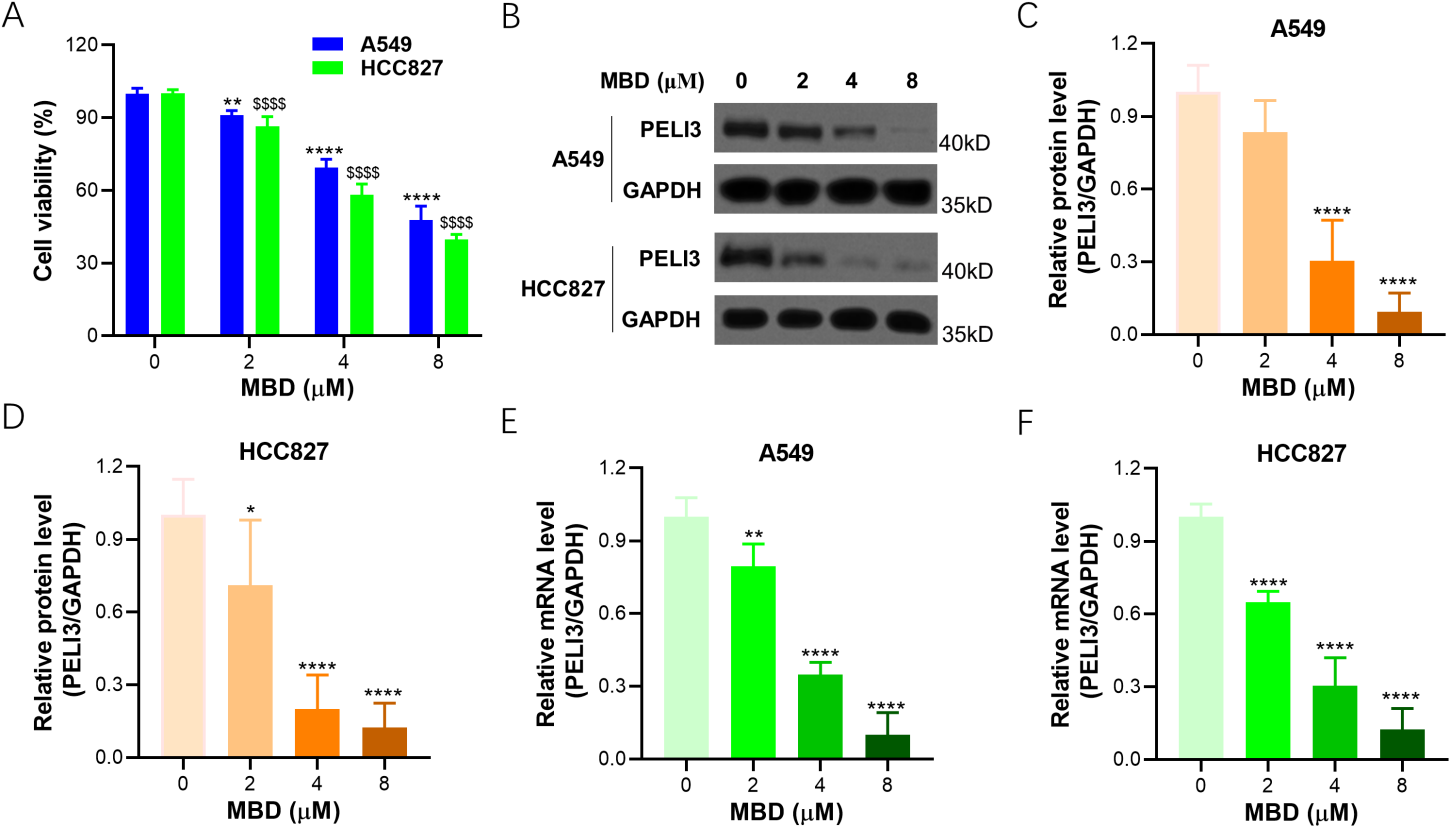
Mebendazole inhibits the expression of PELI3 in NSCLC cells. **(A)** A549 and HCC827 cells were incubated with increasing doses of Mebendazole (MBD) for 24 hours, followed by a CCK-8 assay. **(B)** A549 and HCC827 cells incubated with indicated concentrations of Mebendazole for 24 hours were prepared for immunoblotting analysis against PELI3 and GAPDH. Optical density analysis of PELI3 from **(B)** in A549 **(C)** and HCC827 cells **(D)**. Above cells were also prepared for RT-qPCR analysis to assess the mRNA levels of PELI3 in A549 **(E)** and HCC827 cells **(F)**. GAPDH was used as an internal control. The concentration ‘0’ represented the solvent control, where an equal volume of solvent was added without MBD. ^*^p < 0.05; ^**^p < 0.01; ^****^p < 0.0001; ^$$$$^*p* < 0.0001.

To reveal the treatment benefit of Mebendazole in NSCLC, A549 and HCC827 cells were incubated with Mebendazole, increasing concentration of Mebendazole could decrease the viability of A549 and HCC827 cells in a dose-dependent manner (**Figure 4A**). Immunoblotting was performed in Mebendazole-incubated A549 and HCC827 cells (**Figure 4B**), and decreased PELI3 protein expression was observed in A549 (**Figure 4C**) and HCC827 cells (**Figure 4D**) in a dose-dependent manner. At the same time, decreased PELI3 gene expression was observed in A549 (**Figure 4E**) and HCC827 cells (**Figure 4F**) in an Mebendazole dose-dependent manner.

### Mebendazole increases TRADD protein expression in NSCLC cells

**Figure 5:**
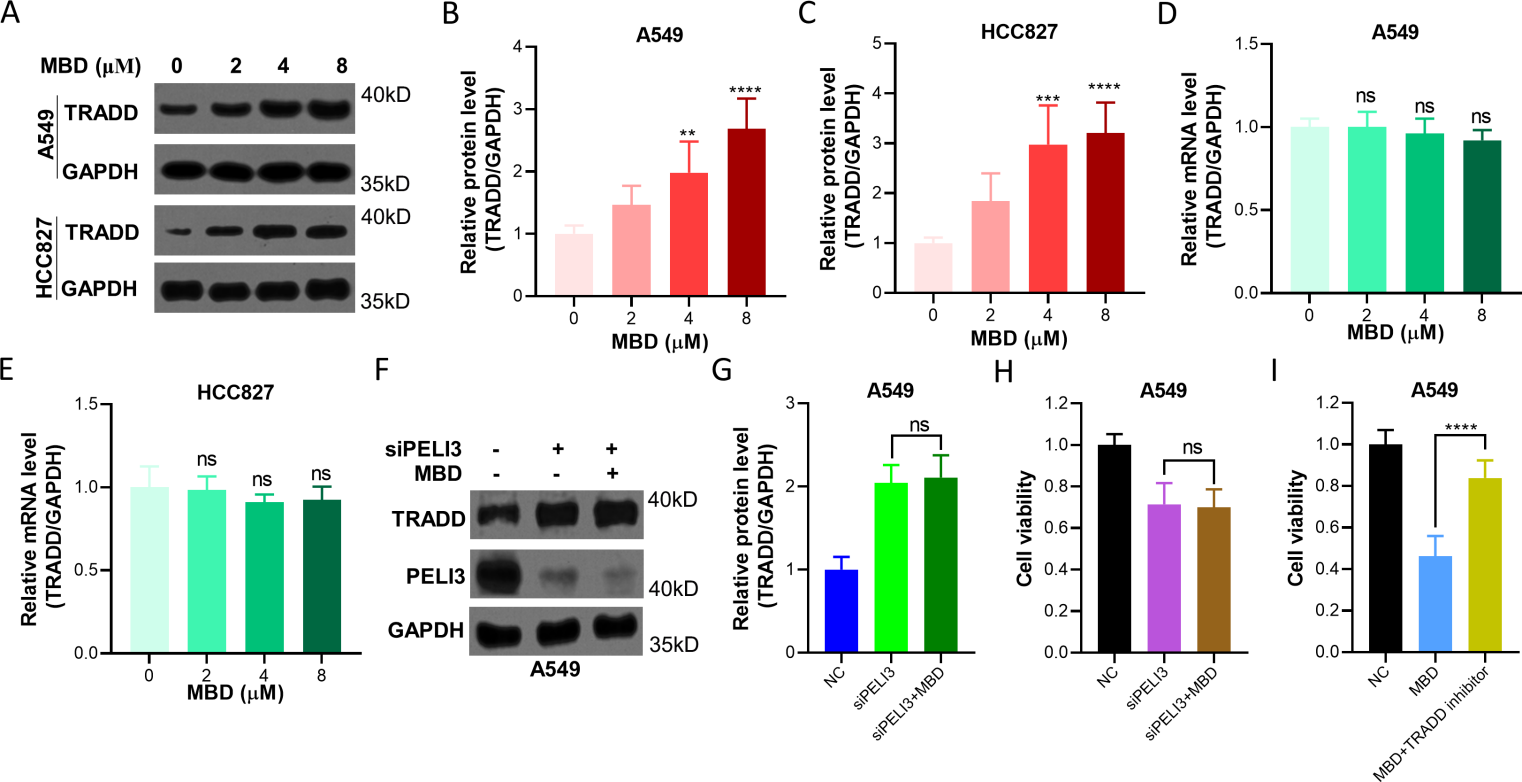
Mebendazole increases the protein expression of TRADD in NSCLC cells. **(A)** A549 and HCC827 cells incubated with indicated doses of Mebendazole (MBD) for 24 hours were prepared for immunoblotting analysis against TRADD and GAPDH. The concentration ‘0’ represented the solvent control, where an equal volume of solvent was added without MBD. Optical density analysis of TRADD from **(A)** in A549 **(B)** and HCC827 cells **(C)**. Above cells were also prepared for RT-qPCR analysis to detect the relative gene expression of TRADD in A549 **(D)** and HCC827 cells **(E)**. GAPDH was used as an internal control. A549 cells were transfected with siNC or siPELI3 for 48 hours, and then cells were incubated with 4 μM MBD for 24 hours, followed by immunoblotting analysis **(F)**, and optical density of TRADD was also measured **(G)**. Above cells were also prepared for CCK-8 assay **(H)**. **(I)** A549 cells were incubated with 8 μM MBD or 1 μM TRADD inhibitor Apostatin-1 for 24 hours, followed by a CCK-8 assay. ^**^p < 0.01; ^***^p < 0.001; ^****^p < 0.0001; ns means not significant.

A549 and HCC827 cells incubated with different concentrations of Mebendazole for 24 hours were prepared for immunoblotting analysis against TRADD and GAPDH (**Figure 5A**), and increased TRADD was observed in A549 (**Figure 5B**) and HCC827 cells (**Figure 5C**) in an Mebendazole dose-dependent manner. At the same time, TRADD gene expression did not show any difference in A549 (**Figure 5D**) and HCC827 cells (**Figure 5E**). A549 cells knocked down of PELI3 were also treated with Mebendazole, but we found that Mebendazole could not significantly increase the protein expression of TRADD in PELI3-silenced cells (**Figures 5F, G**), which further confirmed that Mebendazole stabilized TRADD through regulating PELI3 expression in NSCLC cells. Moreover, we found that Mebendazole did not significantly inhibit the cell viability of NSCLC cells which were knocked down of PELI3 (**Figure 5H**). In addition, A549 cells were incubated with Mebendazole or TRADD inhibitor, and the results showed that the TRADD inhibitor could significantly abolish the cytotoxicity of Mebendazole on NSCLC cells (**Figure 5I**). Above results further suggested that Mebendazole exerted its anti-NSCLC effect through PELI3/TRADD axis.

## Discussion

Drug repurposing attracts much attention in cancer research. Mebendazole, a well-known anti-helminthic drug, has been tested with anti-tumor properties against colorectal cancer, adrenocortical cancer, melanoma, and glioma (11, 17–19). In cell lines of lung cancer, Mebendazole can induce a dose- and time-dependent apoptotic response (20). What’s more, Mebendazole can inhibit tumor growth in the subcutaneous tumor model of the H460 cell (NSCLC cell line) and prohibit lung metastasis formation in the A549 cell line-tail vein injection model (17). For the first time, we test the E3 ubiquitin ligase function of PELI3 against TRADD. We also find that Mebendazole could inhibit the relative expression of PELI3 and promote the relative expression of TRADD, *via* stabilizing it from ubiquitination-mediated degradation. On the other hand, decreased cellular viability is also observed in Mebendazole-incubated NSCLC cells. Here we demonstrate that Mebendazole displays a potent anti-NSCLC effect by inhibiting the E3 ubiquitin ligase function of PELI3.

Initially discovered as the interleukin-1 receptor-associated kinase (IRAK)-interacting proteins, PELI3 can catalyze the ubiquitylation of IRAK1 with E3 ubiquitin ligase function (21). This study further shows that PELI3 can function as an E3 ubiquitin ligase against FADD. PELI3 is aberrantly up-regulated in NSCLC, and PELI3-deficiency could significantly inhibit cell proliferation, migration, and invasion processes. The relative expression of PELI3 can be utilized to predict the prognosis of NSCLC patients (22). On the other hand, PELI3 is cytoprotective in response to the TNF challenge, as it targets receptor-interacting protein 1 kinase and impairs the formation of the death-inducing signaling complex (15). All of these indicate the complexity of the PELI3-mediated signal in NSCLC.

TRADD can function as a scaffold protein to recruit further adaptor proteins to induce two major TNF-induced responses, NF-κB activation and apoptosis (23, 24). In other words, TRADD mediates a direct regulator of both NF-κB-stimulating (TNF-R1–TRADD–RIP/TRAF2) receptor complexes and pro-apoptotic receptor complexes (TNF-R1–TRADD–FADD) (25, 26). Mechanically, TRADD is dispensable for TRAIL-induced apoptosis but is redundant for TNFR1-induced apoptosis upon TNFα stimulation (27). It is worth noting that, the nuclear form of TRADD might act as tumor suppressor to prevent ubiquitination and degradation of isoform p19ARF/ARF of CDKN2A, and disrupt the interaction between thyroid hormone receptor interactor 12 and isoform p19ARF/ARF of CDKN2A (28). All of these indicate the challenge of targeting TRADD in NSCLC.

Some limitations should be indicated. In this study, we demonstrated that PELI3 can function as an E3 ubiquitin ligase to regulate TRADD, and confirmed their physical interaction. However, the specific protein domains mediating the PELI3-TRADD interaction remain to be fully elucidated, which represents a limitation of our current work. While our Co-IP results strongly suggest an association, definitive evidence for a direct physical interaction through *in vitro* binding assays with purified proteins is yet to be obtained. Accordingly, domain mapping using a series of deletion mutants for both PELI3 and TRADD will be an essential focus of our future investigations to delineate the exact binding interface and solidify the specificity of this interaction. In addition, this study demonstrated that PELI3 could promote TRADD polyubiquitination in cellular ubiquitination assays. However, due to inherent limitations of cell-based systems, these findings will be further validated through *in vitro* ubiquitination experiments in our future work. Whether other functions of PELI3 are involved in Mebendazole should be also further investigated. Although our findings demonstrate a clear PELI3-dependent mechanism *in vitro*, several limitations remain regarding *in vivo* applicability. Drug distribution, metabolic stability, and tumor microenvironmental factors may influence Mebendazole’s ability to modulate the PELI3/TRADD axis in living systems. Additionally, off-target effects and differential regulation of ubiquitination pathways *in vivo* may alter therapeutic outcomes. Future studies using xenograft or metastasis models are required to confirm the *in vivo* relevance of these observations. TRADD demonstrates an anti-necroptotic function in RIPK3-expressing HeLa cells, and the death receptor-specific activity of TRADD can be attributed to the hierarchical position in TRAIL and TNFR1-death receptor signaling (27). The potential role of TRADD in inhibiting the viability of NSCLC should be further deciphered. Additionally, it is still unclear how Mebendazole regulates the transcriptional expression of PELI3, and we will continue to investigate the detailed mechanism in our future experiments.

## Conclusions

In summary, Mebendazole is a promising anti-NSCLC drug by inhibiting PELI3 to stabilize TRADD, which may provide a novel therapeutic option for NSCLC patients.

## Data Availability

The original contributions presented in the study are included in the article/supplementary material. Further inquiries can be directed to the corresponding author.
